# Mapping alterations in the local synchrony of the cerebral cortex in schizophrenia

**DOI:** 10.1192/j.eurpsy.2023.2463

**Published:** 2023-10-18

**Authors:** Jesus Pujol, Nuria Pujol, Anna Mané, Gerard Martínez-Vilavella, Joan Deus, Víctor Pérez-Sola, Laura Blanco-Hinojo

**Affiliations:** 1 MRI Research Unit, Department of Radiology, Hospital del Mar, Barcelona, Spain; 2 CIBER de Salud Mental, Instituto de Salud Carlos III, Barcelona, Spain; 3 Institute of Neuropsychiatry and Addictions, Hospital del Mar Research Institute (IMIM), Barcelona, Spain; 4 Department of Clinical and Health Psychology, Autonomous University of Barcelona, Barcelona, Spain; 5 Pompeu Fabra University, Barcelona, Spain; 6 Hospital del Mar Research Institute (IMIM), Barcelona, Spain

**Keywords:** functional connectivity, gamma-aminobutyric acid, parvalbumin, psychosis, somatostatin

## Abstract

**Background:**

Observations from different fields of research coincide in indicating that a defective gamma-aminobutyric acid (GABA) interneuron system may be among the primary factors accounting for the varied clinical expression of schizophrenia. GABA interneuron deficiency is locally expressed in the form of neural activity desynchronization. We mapped the functional anatomy of local synchrony in the cerebral cortex in schizophrenia using functional connectivity MRI.

**Methods:**

Data from 86 patients with schizophrenia and 137 control subjects were obtained from publicly available repositories. Resting-state functional connectivity maps based on Iso-Distant Average Correlation measures across three distances were estimated detailing the local functional structure of the cerebral cortex.

**Results:**

Patients with schizophrenia showed weaker local functional connectivity (i.e., lower MRI signal synchrony) in (i) prefrontal lobe areas, (ii) somatosensory, auditory, visual, and motor cortices, (iii) paralimbic system at the anterior insula and anterior cingulate cortex, and (iv) hippocampus. The distribution of the defect in cortical area synchrony largely coincided with the synchronization effect of the GABA agonist alprazolam previously observed using identical functional connectivity measures. There was also a notable resemblance between the anatomy of our findings and cortical areas showing higher density of parvalbumin (prefrontal lobe and sensory cortices) and somatostatin (anterior insula and anterior cingulate cortex) GABA interneurons in humans.

**Conclusions:**

Our results thus provide detail of the functional anatomy of synchrony changes in the cerebral cortex in schizophrenia and suggest which elements of the interneuron system are affected. Such information could ultimately be relevant in the search for specific treatments.

## Introduction

Substantial research has been conducted to better understand the origin of schizophrenia in the hope of identifying one or few primary factors accounting for most of its varied clinical expression. Succeeding in this effort is important insofar as the identification of selective alterations may well reveal new targets for the development of more specific treatments.

In addition to the advances in dopamine and glutamate neurotransmission research in schizophrenia [1, 2], converging evidence has indicated that the gamma-aminobutyric acid (GABA) interneuron system may be defective at multiple levels of the neuroaxis. Neurophysiological studies have demonstrated GABA system-related deficiencies in the modulation of brainstem reflexes [3, 4], auditory stimuli filtering [5], inhibitory control of corticospinal pathway and prefrontal evoked responses [5, 6], and cortical synchronization [7, 8].

Early post-mortem research suggested a defect in GABA interneurons expressing parvalbumin, which would predominantly implicate the prefrontal cortex [9]. Other studies indicate that the alterations may extend beyond the frontal lobe [10–12] and additionally affect somatostatin interneurons [13]. Nevertheless, the information regarding the anatomical distribution of the cortical GABA system defect is incomplete.

In the cerebral cortex, GABA interneuron deficiency is expressed in the form of local neural activity desynchronization [7, 8, 11] and changes in cortical synchrony may be captured using functional connectivity MRI measures [14, 15]. Abundant neuroimaging research has demonstrated alterations in functional connectivity of multiple types at multiple levels in schizophrenia (reviewed in [16–18]), and some studies indeed revealed a local neural uncoupling compatible with inhibitory interneuron deficiency (e.g., [19–21]). However, the anatomy of cerebral cortex changes in local MRI signal synchrony in schizophrenia has not been detailed and the potential relationship with the interneuron system has not been analyzed.

We mapped the functional anatomy of local, short-range synchrony in schizophrenia using a combination of (Iso-Distant Average Correlation [IDAC]) functional connectivity measures that comprehensively inform on the local functional structure of the cerebral cortex [22–24]. Essentially, we expanded well-established MRI measures of local functional connectivity [25–27] by combining connectivity measures across varying distances. Our IDAC measures represent the average functional MRI temporal correlation of a given brain unit, or voxel, with other units located at increasingly separated iso-distant intervals [22]. The multi-distance approach can offer a more detailed functional mapping of the cerebral cortex that proved its efficacy in effectively distinguishing between major classical anatomo-functional cortical areas [22, 23]. It is important to note that variations in local functional connectivity can express activity variations in both principal (pyramidal) neurons and inhibitory interneurons [28, 29], and thus, a context is needed to properly interpret the direction of change.

We have previously characterized the effect of a typical GABA agonist (alprazolam) on local functional connectivity using our mapping approach [30]. The inhibitory agent alprazolam increased local functional connectivity in the cerebral cortex with a notably system-specific pattern. Significant changes were found in prefrontal, motor, somatosensory, auditory, visual, and orbitofrontal areas. A local synchronization effect has also been demonstrated for other GABA agonists [31–34].

We hypothesized that our IDAC measures would be able to detail the repercussions of the GABA system defect on the cerebral cortex of patients with schizophrenia in the form of weaker local functional connectivity. We anticipated that the changes in functional MRI signal synchrony would involve, and most likely not be limited to, the prefrontal cortex and cortical areas processing sensory information. However, it is worth mentioning that functional connectivity MRI does not measure electrical phenomena related to GABA, which typically synchronize neurons at significantly higher frequencies. Rather, we propose that MRI can be optimally used to complement current neurophysiological evidence by accurately mapping the effects on the synchrony of cerebral cortex hemodynamics.

## Methods

### Study populations

Publicly available neuroimaging data from patients with schizophrenia and healthy subjects were obtained from two open-source datasets: 1 – the Center for Biomedical Research Excellence (COBRE) [35], available at the SchizConnect database (http://schizconnect.org), and 2 – the UCLA Consortium for Neuropsychiatric Phenomics LA5c Study [36], available at the OpenNeuro web platform (https://openneuro.org) under the accession number ds000030.

In both studies, the clinical diagnosis of schizophrenia was established following the Diagnostic and Statistical Manual of Mental Disorders, Fourth Edition-Text Revision (DSM-IV-TR; American Psychiatric Association, 2000) [37], and was based on the Structured Clinical Interview for DSM-IV Axis-I Disorders [38]. Subjects were excluded if they had a history of neurological disorders including head trauma with loss of consciousness, mental retardation, history of substance abuse or dependence (except for nicotine) within the past year, or contraindications to scanning (e.g., claustrophobia, metallic implants). Additional exclusion criteria for healthy volunteers included a current or past psychiatric disorder (except for one lifetime major depressive episode). All subjects had a negative toxicology screen for drugs of abuse at the start of the study. Stable medications were permitted for the patients.

Each study was approved by the corresponding local ethics committees or institutional review board [35, 36]. All participants provided written informed consent according to the corresponding institutional guidelines.

A total of 124 patients with both functional and structural MRI (74 from COBRE and 50 from UCLA datasets) were available in the repositories. Eighty-six patients (71 males and 15 females) were included in the present study based on the availability of functional MRI exams of optimal quality, defined as those free from acquisition artifacts and consisting of at least 80% of volumes after scrubbing. From the control subject repository pool of 213 cases (91 COBRE and 122 UCLA), 137 optimal-quality functional MRI exams were included in the analysis (see quality control in the Supplementary Material). The control sample contained all the available control males with optimal exams (*n* = 111) and a group of 26 females randomly selected to make patients and controls comparable as to sex distribution. The characteristics of the study sample are reported in Table 1.

**Table 1.** Demographic and clinical characteristics of the samples

	86 patients (COBRE = 45 and UCLA = 41)	137 controls (COBRE = 60 and UCLA = 77)
Age, years	35.4 ± 11.6	35.4 ± 10.5
Sex, M/F	71/15	111/26
Handedness, R/L/Amb.	80/5/1	131/3/3
Age at symptoms onset, years^a^	20.4 ± 7.9	
Symptom severity^b^		
PANSS positive	15.7 ± 5.0	
PANSS negative	15.3 ± 4.9	
Medication, *n* ^c^		
Antipsychotics typical	6	
Antipsychotics atypical	69	
Ant. typical and atypical	5	
Benzodiazepines	17	

PANSS, Positive and Negative Syndrome Scale.

*Note: Values are expressed as mean ± standard deviation. Patients and controls did not significantly differ as to mean age and the distribution of sex and handedness.*

a Data available for 45 out of 86 patients.

b From PANSS (*n* = 37) or converted from SAPS/SANS to PANSS (*n* = 41) using the method of van Erp et al. [42].

c Data available for 80 out of 86 patients.

### Assessment of symptom severity

Symptom severity in patients was assessed using the Positive and Negative Syndrome Scale (PANSS) [39] in the COBRE study and the Scale for the Assessment of Negative/Positive Symptoms (SANS) [40] and (SAPS) [41] in the UCLA study. To harmonize symptom variables, SANS and SAPS total scores were converted to PANSS negative and positive subscale scores, respectively, following procedures by van Erp et al. [42].

### MRI acquisition

We selected two studies with highly similar image acquisition procedures to optimize data harmonization across sites. All functional MRI images were collected on 3-Tesla Siemens Trio scanners (Siemens, Erlangen, Germany) using a conventional single-shot, gradient-echo planar imaging sequence. Acquisition parameters in the COBRE study were set as repetition time, 2000 ms; echo time, 29 ms; pulse angle, 75°; 24-cm field of view; 64 × 64-pixel matrix; and slice thickness of 3.5 mm (slice gap, 1.05 mm). Thirty-three sequential sections, parallel to the anterior–posterior commissure line, were acquired to generate 150 whole-brain volumes (total duration of 5 min), excluding 2 initial additional dummy volumes. The parameters in the UCLA study were set as repetition time, 2000 ms; echo time, 30 ms; pulse angle, 90°; 19.2-cm field of view; 64 × 64-pixel matrix; and slice thickness of 4 mm. Thirty-four sections were acquired to generate 152 whole-brain volumes (total duration of 5 min 4 s). Participants were asked to remain relaxed and keep their eyes open throughout. 3D anatomical images were also obtained in each case based on a high-resolution T1-weighted three-dimensional magnetization-prepared rapid gradient-echo (MPRAGE) sequence, which served to assist functional connectivity image processing.

### IDAC maps

Imaging data were processed using MATLAB version 2016a (The MathWorks Inc, Natick, MA) and Statistical Parametric Mapping software (SPM12; The Wellcome Department of Imaging Neuroscience, London). Image processing steps adopted to generate the cerebral cortex IDAC maps have been previously reported [22] and a detailed description is provided in the Supplementary Material. Below is a summary.

Functional MRI images were slice-time corrected, realigned, co-registered to their corresponding anatomical image, re-sliced to 3 × 3 × 3 mm resolution, and smoothed by convolving the image with a 4 × 4 × 4 mm full width at half maximum Gaussian kernel. Motion-affected image volumes were discarded using conventional scrubbing procedures [43].

IDAC measures were then estimated in native space. The computation was conducted in a gray matter mask split into left and right hemispheres. Whole-cortex IDAC maps were generated by estimating the average temporal correlation of each voxel with all its neighboring voxels placed at increasingly separated Euclidean iso-distant intervals (definition and mathematical formulation is provided in the Supplementary Material). Three IDAC maps were obtained at distance intervals 5–10, 15–20, and 25–30 mm. The analyses were adjusted by including six rigid body realignment parameters, their first-order derivatives, average white matter, Cerebrospinal fluid (CSF), and global brain signal as regressors. All functional MRI time series were band-passed with a discrete cosine transform filter letting through frequencies in the 0.01–0.1 Hz interval.

Finally, the resulting IDAC maps in native space were normalized to the Montreal Neurological Institute (MNI) space with a back-transformation process, that is, individual 3D anatomical images had previously been segmented and registered to the MNI space and the inverse deformation fields provided by SPM in this step were applied to the IDAC maps.

**Figure 1.**
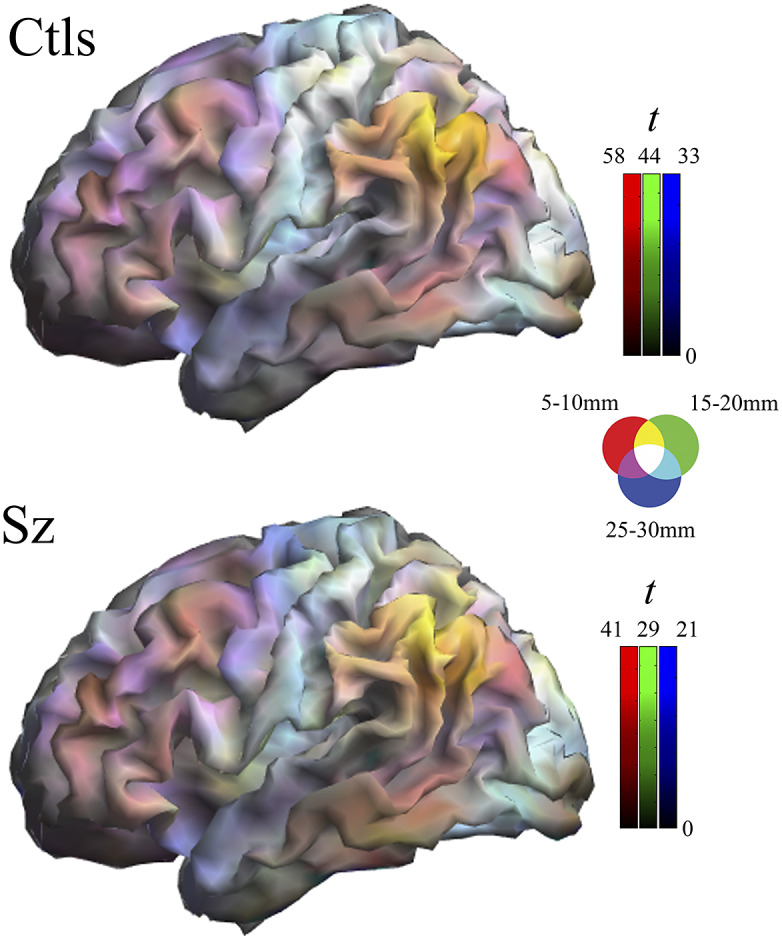
Composite one-sample Iso-Distant Average Correlation (IDAC) brain maps. The images show the result of superimposing the three IDAC maps using an RGB (red, green, and blue) color display. Note that such multi-distance maps are able to discriminate between various cortical areas. Ctls, control subjects; Sz, schizophrenia.

Multi-distance IDAC color maps were obtained from the overlay of the three IDAC maps using an RGB color codification (see Figure 1). RGB color channels enabled the display of three values simultaneously, RED corresponding to the results from 5 to 10 mm IDAC map analyses, GREEN from 15 to 20 mm, and BLUE from 25 to 30 mm. The overlapping of these primary colors produces a full range of secondary colors. Composite RGB maps were generated from one-sample t-maps obtained for each distance in both study groups and from the between-group comparison t-maps.

### Statistical analysis

IDAC connectivity maps were included in SPM group-wise random-effects analyses adopting a 2 × 3 mixed design ANOVA (ANCOVA) model (i.e., group [patients, controls] by distance [5–10, 15–20, and 25–30 mm]). A motion summary measure (mean inter-frame motion [43]) for each participant was included as a covariate in all analyses. We specifically tested for group effects to map cortical areas with altered connectivity (primary study question) and for group-by-distance interactions to determine whether the alterations concerned the spatial structure (i.e., differential implication of distinct local distances). In all analyses, results were considered significant when clusters formed at a threshold of *p* < 0.005 survived whole-brain family-wise error correction (*p* < 0.05), calculated using SPM.

## Results

One-sample maps of cerebral cortex functional connectivity were generated for the three local distances and the outputs are presented together using RGB display. Figure 1 and Supplementary Figure 1 illustrate the extent to which the human cerebral mantle is functionally heterogeneous in these measures. Distinct anatomo-functional areas show a different functional structure determined by variations in the relative strength of connectivity at locally short, locally middle, and locally long distances. Cortical area differentiation is evident in both control subjects and patients with schizophrenia. However, as can be appreciated upon visual inspection, the maps are not identical.

**Figure 2.**
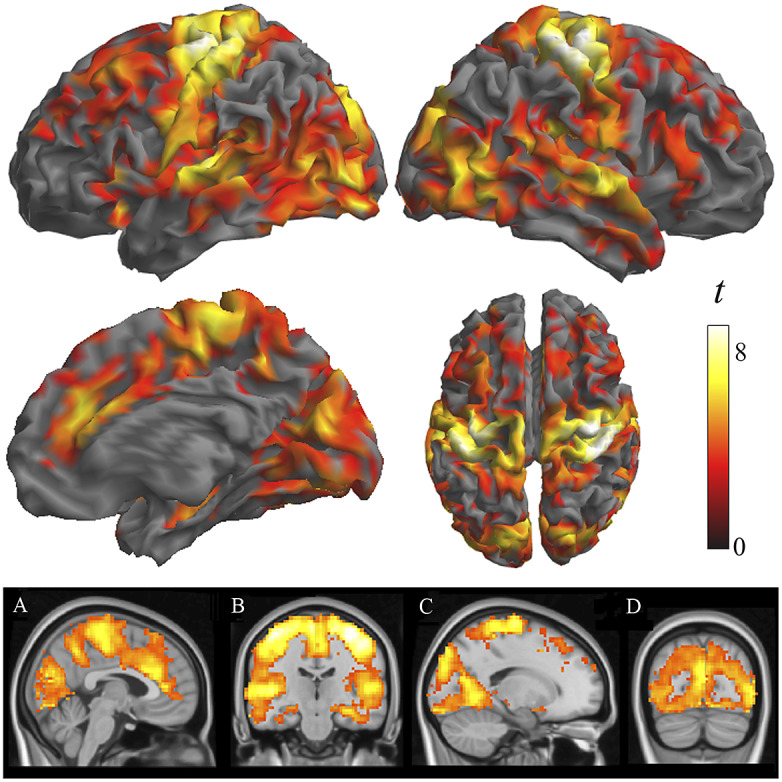
Differences between patients with schizophrenia and control subjects in IDAC measures across the three distance maps. The images show ANOVA results in the direction of patients showing weaker local functional connectivity (negative effect of group across distances). Orthogonal displays (bottom images) are shown to detail the implication of the anterior cingulate cortex (A), the hippocampus (B), and the relative preservation of the occipital pole (C and D).

Two-sample analyses confirmed that both groups were significantly different in local functional connectivity. Supplementary Table 1 reports the results from ANOVA showing group differences across the three distances. All the identified group differences were in the direction of patients showing weaker local functional connectivity (i.e., lower functional MRI signal synchrony) (Figure 2). Highly significant changes were bilaterally observed in each sensory cortex, primary motor cortex, insula extending to the frontal operculum and orbitofrontal cortex, anterior cingulate cortex, dorsal prefrontal cortex, and hippocampus. Remarkably, the primary somatosensory cortex was affected in almost its entire extension. The alteration in the auditory cortex was maximal in the primary auditory area. By contrast, the visual cortex showed widespread changes but virtually excluded the occipital pole. Sensitivity analyses separately including sex, age, and scanner as covariates did not reveal any relevant effect (Supplementary Figure 2 and Supplementary Table 2).

**Figure 3.**
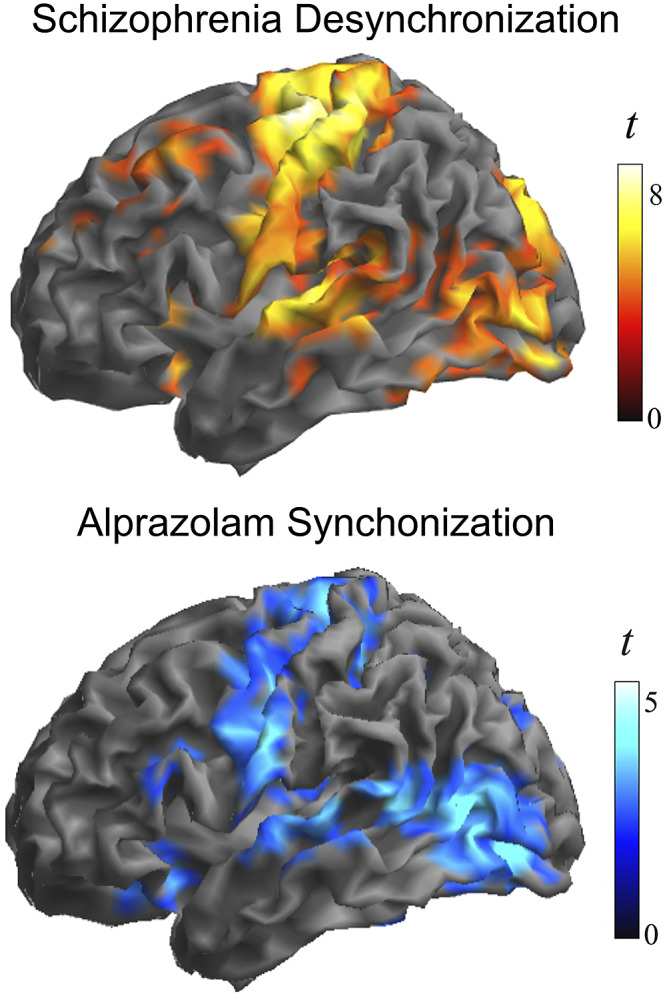
Alterations in the local synchrony of the cerebral cortex in schizophrenia and the cortical synchronization action of GABA inhibition. Top, the identified group differences at a higher threshold (voxel *t* > 4) to emphasize the areas with the largest effect. Bottom, cortical synchronization by the GABA agonist alprazolam observed using identical functional connectivity measures. Adapted, with permission, from Blanco-Hinojo et al. [30].

Figure 3 shows group differences at a higher threshold (voxel *t* > 4) to emphasize the areas with the largest effect. Note the conspicuous cortical area coincidence with the synchronization effect of the GABA agonist alprazolam observed using identical local functional connectivity measures in an early study by our group [30]. That is, patients with schizophrenia at rest showed weaker local synchrony in cortical areas typically synchronized by the GABA agonist alprazolam. The correlation between the corresponding t-maps was high, showing a Person’s *r* of 0.46 (*p* < 0.00001 after controlling for spatial autocorrelation [44]).

**Figure 4.**
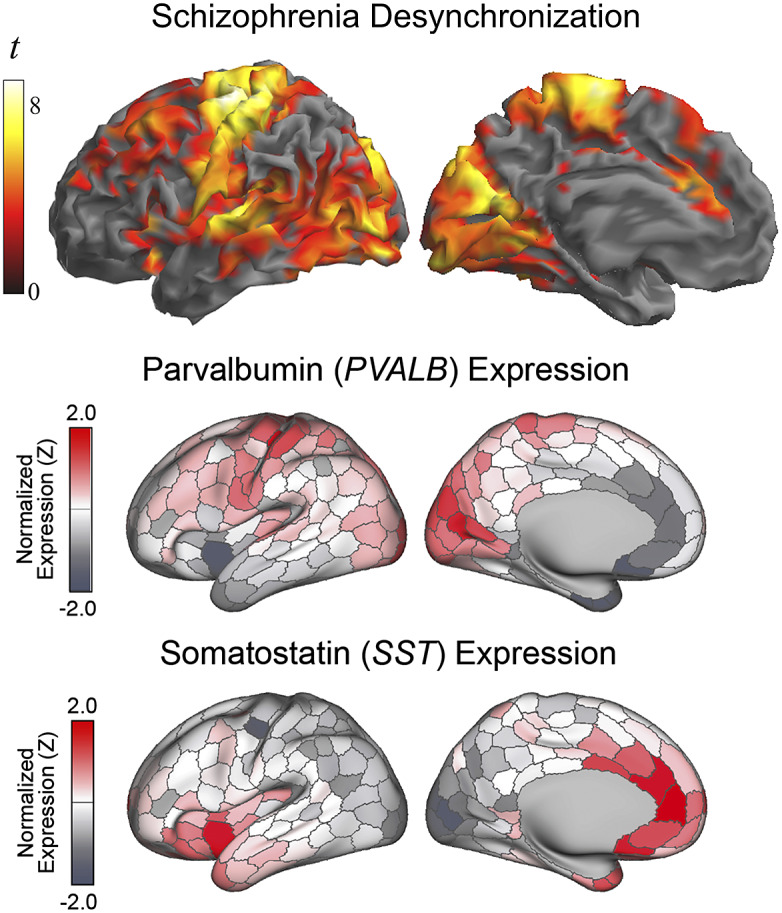
Alterations in the local synchrony of the cerebral cortex in schizophrenia and the distribution of parvalbumin and somatostatin GABA interneurons in humans (adapted, with permission, from Anderson et al. [45]). Differences between patients with schizophrenia and control subjects in IDAC measures (top) are presented as in Figure 1.

The anatomical resemblance between the study results and the cortical GABA system was also notable with the combined distribution of parvalbumin and somatostatin GABA interneurons in humans (summarized in Anderson et al. [45], from the Allen Human Brain Atlas [https://human.brain-map.org/]). The human expression of parvalbumin GABA interneurons is maximal in the motor cortex, somatosensory cortex, auditory cortex, visual areas, and dorsal prefrontal cortex (Figure 4). On the other hand, the expression of somatostatin GABA interneurons is maximal in the anterior insula-orbitofrontal cortex and anterior cingulate cortex. Therefore, the areas most affected in patients with schizophrenia characteristically show a high density of parvalbumin or somatostatin GABA interneurons, which interestingly both developmentally derive from the medial ganglionic eminence of the subpallium [46]. In contrast, the resemblance of our findings to the cortical distribution of GABA interneurons subclasses deriving from the embryonic caudal ganglionic eminence (i.e., VIP, LAMP-5, and SNCG) was minimal, indicating a level of specificity regarding the interneuron type putatively implicated (see Supplementary Figure 3).

Although the weakening in local functional connectivity affected the three measured distances (Supplementary Figure 4), a tendency to a greater effect in long distances was observed in some association cortices and, in short distances, in the sensorimotor cortex and visual areas. However, formally tested group-by-distance interaction was significant only for a restricted area in the sensorimotor cortex (Supplementary Table 1 and Supplementary Figure 5).

**Figure 5.**
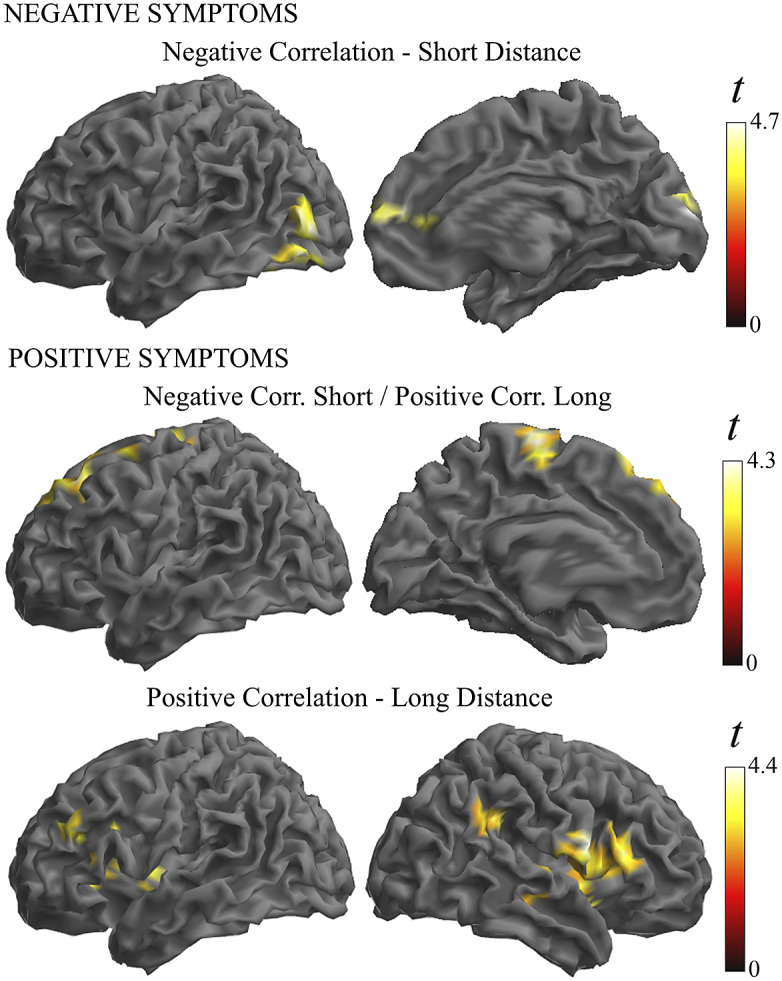
Illustration of the correlation analysis results. Negative symptoms were associated with weaker functional connectivity in the short-distance maps (top images). Positive symptoms were associated with the combination of weaker functional connectivity in the short-distance maps and stronger connectivity in the long-distance maps (middle images). In addition, positive symptoms were associated with a stronger connectivity at long distances (bottom images).

Finally, a regression analysis was conducted in the patient group to establish whether symptom severity was associated with the identified functional connectivity alterations (Supplementary Table 3 and Figure 5). Negative symptoms were associated with a weaker functional connectivity in the anterior cingulate cortex and visual areas in the short- and middle-distance maps. Positive symptom scores did not show a net negative correlation, but instead they were associated with the combination of weaker functional connectivity in the short-distance maps and stronger connectivity in the long-distance map (i.e., correlation interaction across distances) in the motor cortex and prefrontal cortex. In addition, the analysis of individual distances showed a significant association of positive symptoms with stronger connectivity at long distances in the Broca area region and its homologue in the right hemisphere.

## Discussion

We used functional connectivity MRI measures to capture potential alterations in cerebral cortex local synchrony in patients with schizophrenia. Robust changes were identified in several brain areas in the form of weaker functional connectivity compatible with GABA system dysfunction. Importantly, details concerning the anatomical distribution of such changes may provide useful information as to the nature of the inhibitory system defect by further indicating which interneuron types may be predominantly affected.

Patients with schizophrenia showed weaker functional MRI signal synchrony involving distinct cortical domains ranging from the prefrontal cortex to the limbic system. The alterations were evident in the frontal association cortex, each sensory cortex modality and motor cortex, the paralimbic system at the anterior insula and anterior cingulate cortex, and the hippocampus. As for the sensory cortex, the changes were more obvious in somatosensory, visual, and auditory areas. However, regions including the gustatory (insula-frontal operculum) and olfactory (orbitofrontal) cortices were also implicated.

Two datasets served to establish the resemblance between the cortical distribution of our findings and the cortical GABA system. Firstly, we used data from a previous study by our group testing the effect of alprazolam on cerebral cortex local functional connectivity in healthy volunteers [30]. Alprazolam is a GABA agonist with inhibitory action and obvious effects on functional MRI signal synchronization. It is an interesting example of how functional connectivity MRI measures may relate neural inhibition to “paradoxical” increases in functional connectivity. We observed a notable similarity between the synchronization effect of alprazolam and the distribution of the defect in cortical area synchrony in schizophrenia (Figure 3).

The complex GABA system includes a variety of inhibitory interneuron types with different morphology, anatomical distribution, and gene expression [47, 48]. One of the most abundant types of interneurons expresses parvalbumin. A high density of parvalbumin interneurons in humans is found in a few subcortical structures (e.g., thalamus, trigeminal nuclei, and cerebellum) and in prefrontal, somatosensory, visual, auditory, and motor areas of the cortical mantle [45, 49]. The cortical sites we found with altered functional MRI signal synchrony in patients with schizophrenia precisely include the set of cortical areas with high parvalbumin density in humans. The parvalbumin interneuron defects identified in selected areas in post-mortem studies in patients with schizophrenia are also consistent with the anatomy of our findings [9–11].

Also, importantly, a few studies indicate that the alterations may not be limited to the parvalbumin-type interneurons [10, 13]. In our analysis, functional connectivity changes in schizophrenia additionally implicated the areas showing a high density of somatostatin interneurons. We therefore provide novel evidence with a more complete picture of the repercussions of the GABA system dysfunction on the cerebral cortex.

Cortical maps of parvalbumin and somatostatin GABA interneurons in humans are minimally overlapped. Instead, areas showing high parvalbumin interneuron density show low somatostatin interneuron density, and vice versa [45]. Therefore, the influence of both cell lines on brain function needs to be different and complementary. In general, parvalbumin interneurons are most abundant in the neocortex and somatostatin interneurons in paralimbic areas. This is consistent with the fact that the clinical expression of schizophrenia includes symptoms related to both the cognitive and affective domains.

The hippocampus may be an exception to the minimal interneuron-type anatomical overlapping, as it shows a relatively high abundance of parvalbumin and somatostatin interneurons [45, 50]. Consistently, we found altered local functional connectivity in the hippocampus in patients with schizophrenia and post-mortem studies have demonstrated a lower expression of both interneuron types [50, 51].

It is worth noting that the auditory and visual cortices were not affected in the same way in our study. That is, whereas early auditory areas at the Heschl’s gyri were uniformly altered, functional connectivity changes were not evident in the occipital pole. This part of the visual cortex serves central, high acuity vision. In contrast, eccentric areas in the occipital lobe are more involved in holistic and peripheral vision [52–54]. Relevantly, one of the most characteristic perceptive dysfunctions in patients with schizophrenia is instability in the rapid extraction of global information from a visual stimulus, which relies more on global and peripheral vision than on central vision [53, 55–57]. Also, we wonder whether a higher prevalence of auditory as opposed to visual hallucinations in patients with schizophrenia [58] might be related to a different nature of local synchrony alterations in auditory and visual cortices.

The normal differentiation of short-range, local functional connectivity is highly active during adolescence [19] and is sexually dimorphic in some cortical areas showing a synchrony defect in schizophrenia in the present study (i.e., sensorimotor cortex, visual cortex, and prefrontal cortex). Specifically, boys physiologically appear to require a lower maturational reduction in local functional connectivity in such areas during the transition from childhood to adulthood [24]. Therefore, it is possible that the risk of developing schizophrenia in this critical period and the higher incidence in males [2] could to some extent be related to the effect of environmental stressors on cortical inhibitory interneurons, presumably via promoting excessive synaptic pruning [2, 19, 59].

In our correlation analysis, weaker functional MRI signal synchrony was coherently associated with the severity of schizophrenia symptoms in a part of the altered cortical areas. In the visual cortex and anterior cingulate cortex, higher negative symptom scores predicted weaker functional connectivity, and higher positive symptoms predicted a combination of weaker functional connectivity at short distances and stronger functional connectivity at long distance in the motor cortex and prefrontal cortex. These are relevant results emphasizing the functional significance of the observed alterations in the cerebral cortex in patients with schizophrenia.

We also observed a positive correlation between symptom severity and functional connectivity measures. Positive symptoms of schizophrenia predicted higher synchrony, particularly in the Broca area region and its homologue in the right hemisphere. Importantly, significant correlations were observed only for long distances. This association may be more directly interpreted as indicative of relatively distant synchronization effects stemming from the activity of principal (pyramidal) neurons rather than being a distinct expression of local inhibitory interneuron alterations. Positive symptoms of schizophrenia (e.g., the experience of hallucinations) are associated with cortical hyperactivity [60, 61] and increased functional connectivity [62]. Thus, for such an association, stronger functional connectivity could better express the increase in the number of co-activated principal neurons. Our finding may be of interest in the debate on the participation of language-related areas in the generation of auditory hallucinations [61, 62].

An important limitation of our study concerns to the medication status of patients. All patients were taking one or more drugs. Antipsychotics and benzodiazepines have a demonstrated effect on neural inhibition [30, 63]. Therefore, our functional connectivity measures may be sensitive to the effect of schizophrenia treatments. However, medication in our study may more likely have attenuated differences in functional connectivity between patients and controls rather than causing them. Indeed, antipsychotic agents, particularly atypical antipsychotics, reduce differences between patients and controls in terms of the neurophysiological measures of neuronal inhibition deficit [3, 63] and can restore the expression of parvalbumin in experimentally altered GABA interneurons [e.g., 48, 64, 65].

In conclusion, we used an imaging approach to map the local functional structure of the cerebral cortex in patients with schizophrenia and identified alterations in functional MRI signal synchrony compatible with a GABA system defect. Robust changes were observed in prefrontal lobe areas and sensory cortices showing high density of parvalbumin-expressing interneurons in humans. Functional connectivity alterations also implicated paralimbic areas showing a high density of somatostatin-expressing interneurons. Our results thus provide novel details regarding the functional anatomy of the local synchrony defect at the cerebral cortex and suggest which elements of the inhibitory interneuron system are affected. This information could ultimately be relevant in the search for specific treatments with the aim of improving the symptoms of schizophrenia without affecting global brain function.

## Supporting information

Pujol et al. supplementary material Pujol et al. supplementary material

Pujol et al. supplementary material Pujol et al. supplementary material

## Supplementary material

The supplementary material for this article can be found at https://doi.org/10.1192/j.eurpsy.2023.2463.
