# Supplementary material for: Mapping alterations in the local synchrony of the cerebral cortex in schizophrenia
Source: Eur Psychiatry. 2023 Oct 18;66(1):e84. doi: 10.1192/j.eurpsy.2023.2463 (PMC10755567; doi:10.1192/j.eurpsy.2023.2463)
Supplement: Pujol et al. supplementary material [file S092493382302463Xsup001.pdf]

Mapping alterations in the local synchrony of the cerebral cortex in schizophrenia

Supplementary Material

Supplementary Methods

Supplementary Figures

Supplementary Tables

Supplementary Methods

Iso-Distant Average Correlation (IDAC) maps

A novel mapping was used to characterize the functional structure of the cerebral cortex based on Iso-Distant Average Correlation (IDAC) measures. Essentially, IDAC mapping expands well-established MRI measures of local functional connectivity (Sepulcre et al. 2010; Tomasi and Volkow 2010; Zang et al. 2004) by combining the connectivity maps of varying distances. Composite IDAC maps may uniquely inform the connectivity-related specialization of the cerebral cortex as local connectivity is distance-specific to a large extent and proved to discriminate well between major classical anatomo-functional cortical areas (Macià et al., 2018; Pujol et al., 2019).

Image processing

Imaging data were processed using MATLAB version 2016a (The MathWorks Inc, Natick, Mass) and Statistical Parametric Mapping software (SPM12; The Wellcome Department of Imaging Neuroscience, London).

Anatomical and functional images were visually inspected to detect possible acquisition artifacts. Functional MRI images were slice-time corrected, realigned to their mean, co-registered to their anatomical images with an affine transformation, re-sliced to 3x3x3 mm and then smoothed by convolving the image with a 4x4x4mm3 full width at half maximum (FWHM) Gaussian kernel.

Quality control. The resulting parameters from the realignment step were used for scrubbing, namely, discarding motion-affected volumes (Power et al., 2014). For each subject, mean inter-frame motion measurements (Pujol et al., 2014) served as an index of data quality to flag volumes of suspect quality across the run. At time points with mean inter-frame motion > 0.3 mm, the corresponding volume, the immediately preceding and the succeeding two volumes were all discarded. Subjects showing less than 80% of volumes (4 min) after scrubbing were not included in the functional MRI analysis. A total of 38 patients (27 out of 98 males and 11 out of 26 females) and 29 control subjects (19 out of 130 males and 10 out of 83 females) were primarily discarded according to this image quality criterion.

A warping matrix was also estimated for every subject to match the Montreal Neurological Institute (MNI) space using segmentation of individual 3D anatomical images via SPM. The

inverse deformation fields to the MNI space were applied to the IDAC results to enable group inferences.

IDAC computations (see below) were conducted in a gray matter mask split into left and right hemispheres, so that no adjacent voxels from the medial regions of one hemisphere would be locally associated with those from the other hemisphere. The two hemispheres were brought back together once the IDAC values had been calculated. The left and right hemisphere gray matter masks were obtained by setting a threshold of $p>0.4$ on the gray matter probability maps obtained from SPM. As IDAC value estimations were carried out in every subject's native space, the template masks were back-transformed with the inverse estimated normalization.

In IDAC computation analyses, all time series were regressed on the 6 rigid body realignment parameters and their first-order derivatives, and on the average white matter, CSF and global brain signals extracted from the native tissue masks. Finally, all functional MRI time series were band-passed with a Discrete Cosine Transform (DCT) filter letting through frequencies in the 0.01-0.1Hz interval.

Whole-cortex IDAC maps were generated by estimating the average temporal correlation of each voxel with all its neighboring voxels placed at increasingly separated Euclidean iso-distant intervals. Three IDAC maps were obtained at distance intervals 5-10mm, 15-20mm and 25-30mm.

Multi-distance IDAC color maps were obtained from the overlay of the three IDAC maps using an RGB color codification (see Figures). RGB color channels enabled the display of three values simultaneously. RED corresponding to the results from 5-10mm IDAC map analyses, GREEN from 15-20mm and BLUE from 25-30mm. The overlapping of these primary colors produces a full range of secondary colors.

Definition of Iso-Distant Average Correlation (IDAC)

We defined the concept of "Iso-Distant Average Correlation" (IDAC) to describe the pattern of correlation decay in the close vicinity of a voxel (Macia et al., 2018). $IDAC_i(h)$ was consequently defined as the average temporal correlation of voxel *i* with all the voxels located at a given Euclidean distance interval *h*. Functional MRI data sets being a discrete sample, any distance interval h must be necessarily transformed into a discrete iso-distant interval $H_k=(h_k, h_{k+1})$, with

h_k being a set of successively increasing distances covering the whole vicinity of a given voxel (see Figure).

The set of iso-distant intervals H_k were selected so that temporal correlations were mainly positive (Macià et al., 2018), decreased monotonically and in which horizontal axon collaterals were considered likely to form local networks. For the present study, we defined 3 iso-distant intervals: 5-10mm, 15-20mm and 25-30mm, with constant thicknesses but increasing number of voxels.

We first computed a correlation matrix *C* of Pearson coefficients comparing the functional MRI signal time course of all the voxels in our study mask with each other's.

$$C_{i,j} = \frac{\sum_{k=1}^{M}\left(Y_{i,k} - \overline{Y_i}\right) \cdot \left(Y_{j,k} - \overline{Y_j}\right)}{\sqrt{\sum_{k=1}^{M}\left(Y_{i,k} - \overline{Y_i}\right)^2} \cdot \sqrt{\sum_{k=1}^{M}\left(Y_{j,k} - \overline{Y_j}\right)^2}}$$

where M is the length of the functional MRI signal time series and *i* and *j* index all the voxels entering our study mask. We then transformed the Pearson correlation matrix *C* into a Gaussian distributed z-score correlation matrix Z by applying a Fisher transform.

$$Z_{i,j} = \frac{\sqrt{M-3}}{2} \cdot \ln\left(\frac{1 + C_{i,j}}{1 - C_{i,j}}\right)$$

We obtained then $\mathrm{IDAC}_i(h_k)$ by averaging the correlation coefficients of voxel *i* with all the voxels *j* belonging to the interval H_k.

$$IDAC_i(h_k) = \frac{\sum_{j \epsilon H_{k,i}} Z_{i,j}}{N_{k,i}}$$

In short, IDAC values are defined as the mean correlation z-score between one voxel's functional MRI signal and the functional MRI signal of all the voxels within the iso-distant interval $H_{k,i}$. Note that, for a given distance interval *k*, the number of voxels within the concentric iso-distant interval $N_{k,i}$ is not necessarily the same for every voxel *i* due to the edge effects of the study mask.

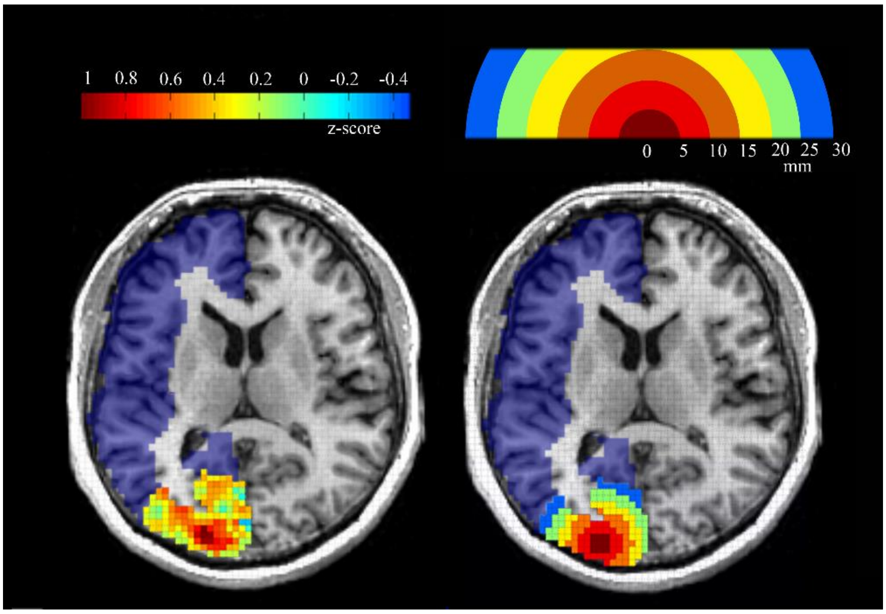

Figure. fMRI Temporal correlations between one voxel ("seed") and its neighboring peripheries present a characteristic decreasing spatial gradient. LEFT: Fisher-transformed z-scores of a correlation map with a "seed" voxel in the visual area from a single subject. Voxel resolution is 3x3x3mm and results are constrained to distance lags h_k<30mm and within the subject's native gray-matter mask (blue shade). RIGHT: Six different Iso-distant intervals as they are used to calculate different IDAC values in our study.

Supplementary References

Macià D, Pujol J, Blanco-Hinojo L, Martínez-Vilavella G, Martín-Santos R, Deus J. Characterization of the spatial structure of local functional connectivity using multi-distance average correlation measures. Brain Connectivity 2018 Jun;8(5):276-287.

Power JD, Mitra A, Laumann TO, Snyder AZ, Schlaggar BL, Petersen SE. Methods to detect, characterize, and remove motion artifact in resting state fMRI. Neuroimage. 2014 Jan 1;84:320-41.

Pujol J, Macià D, Blanco-Hinojo L, Martínez-Vilavella G, Sunyer J, de la Torre R, Caixàs A, Martín-Santos R, Deus J, Harrison BJ. Does motion-related brain functional connectivity reflect both artifacts and genuine neural activity? Neuroimage. 2014 Nov 1;101:87-95.

Pujol J, Blanco-Hinojo L, Maciá D, Alonso P, Harrison BJ, Martínez-Vilavella G, Deus J, Menchón JM, Cardoner N, Soriano-Mas C. Mapping alterations of the functional structure of the cerebral cortex in obsessive-compulsive disorder. Cereb Cortex. 2019 Dec 17;29(11):4753-4762.

Sepulcre J, Liu H, Talukdar T, Martincorena I, Yeo BT, Buckner RL. The organization of local and distant functional connectivity in the human brain. PLoS Comput Biol. 2010;6(6):e1000808.

Tomasi D, Volkow ND. Functional connectivity density mapping. Proc Natl Acad Sci U S A. 2010;107(21):9885-9890.

Zang Y, Jiang T, Lu Y, He Y, Tian L. Regional homogeneity approach to fMRI data analysis. Neuroimage. 2004;22(1):394-400.

Supplementary Figures

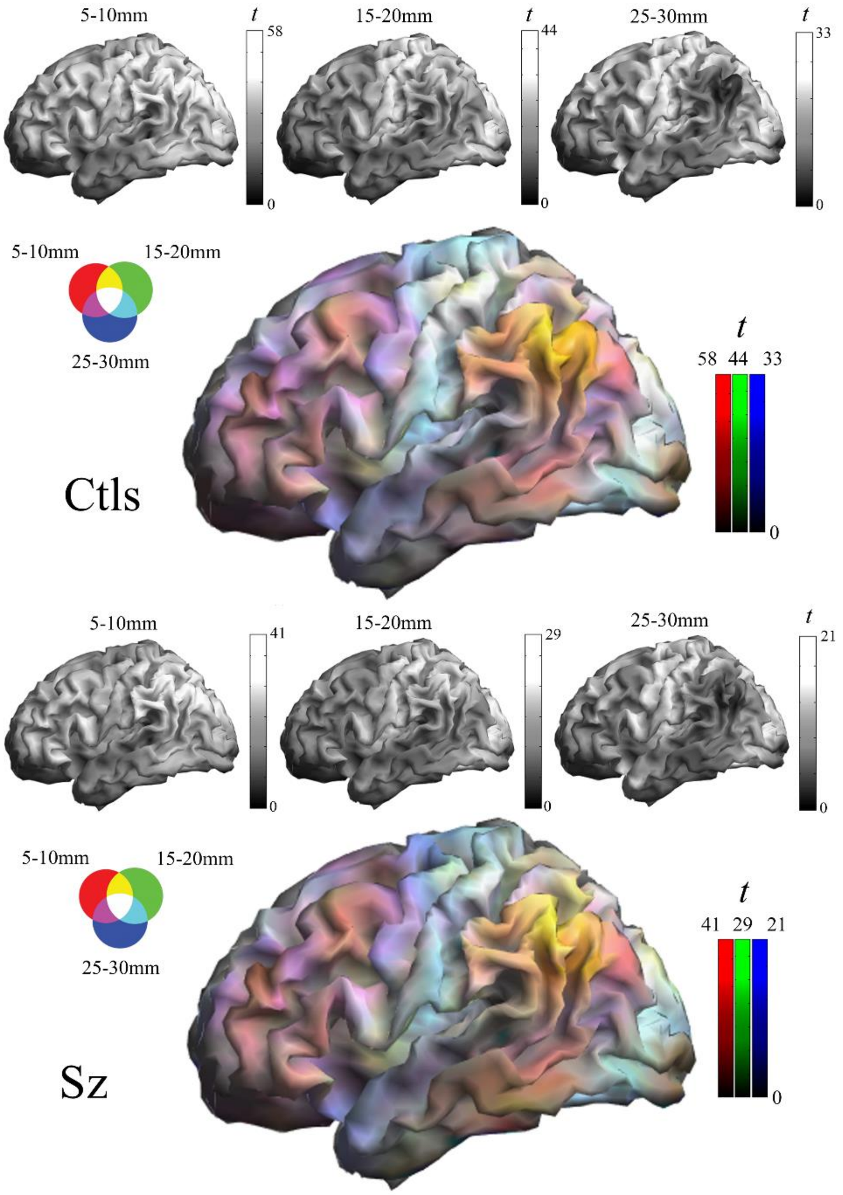

Supplementary Figure 1. One-sample Iso-Distant Average Correlation (IDAC) brain maps. The gray images correspond to individual distance IDAC maps. The color images show the result of superimposing the three IDAC maps using RGB (red, green and blue). The composite images are thus made up of primary RGB colors and their secondary combinations. Ctls, control subjects. Sz, schizophrenia.

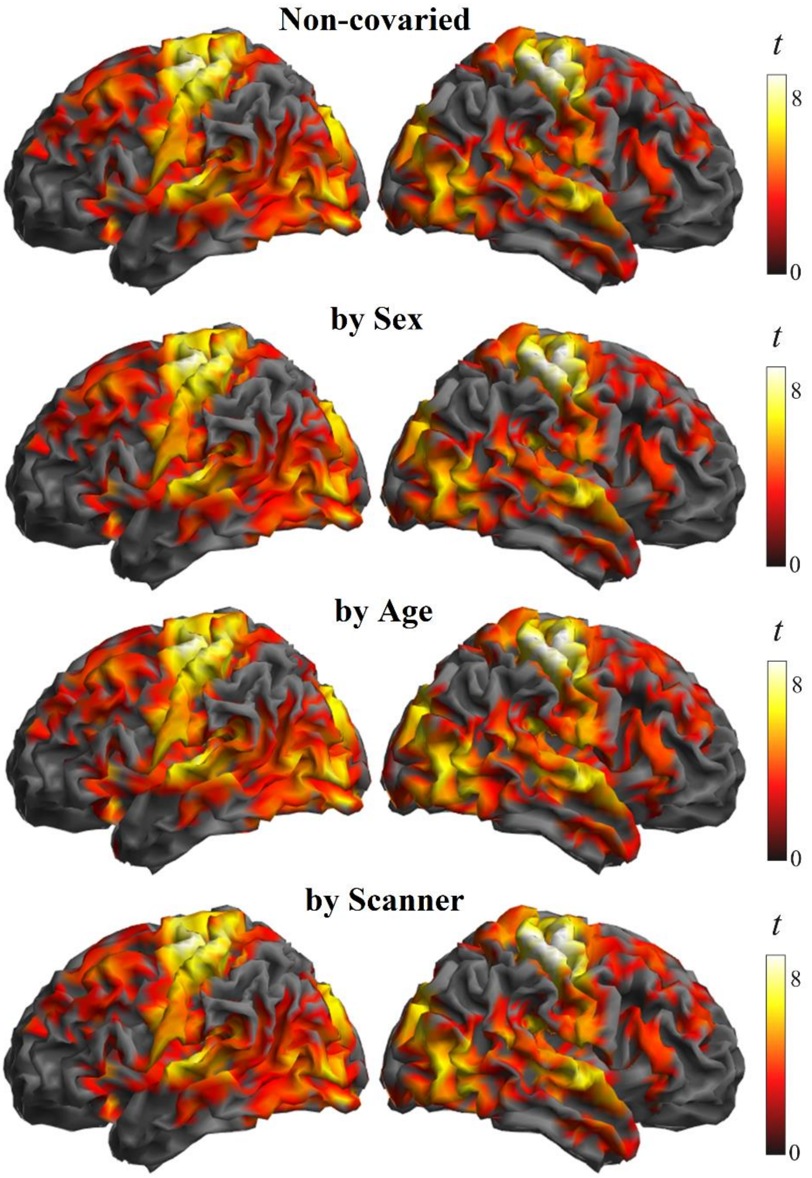

Supplementary Figure 2. Differences between patients with schizophrenia and control subjects in IDAC measures across the three distance maps with and without the inclusion of sex, age and scanner as covariates. The images show ANOVA results in the direction of patients showing weaker local functional connectivity (negative effect of group across distances). These analyses did not reveal any relevant effect on the anatomy of changes.

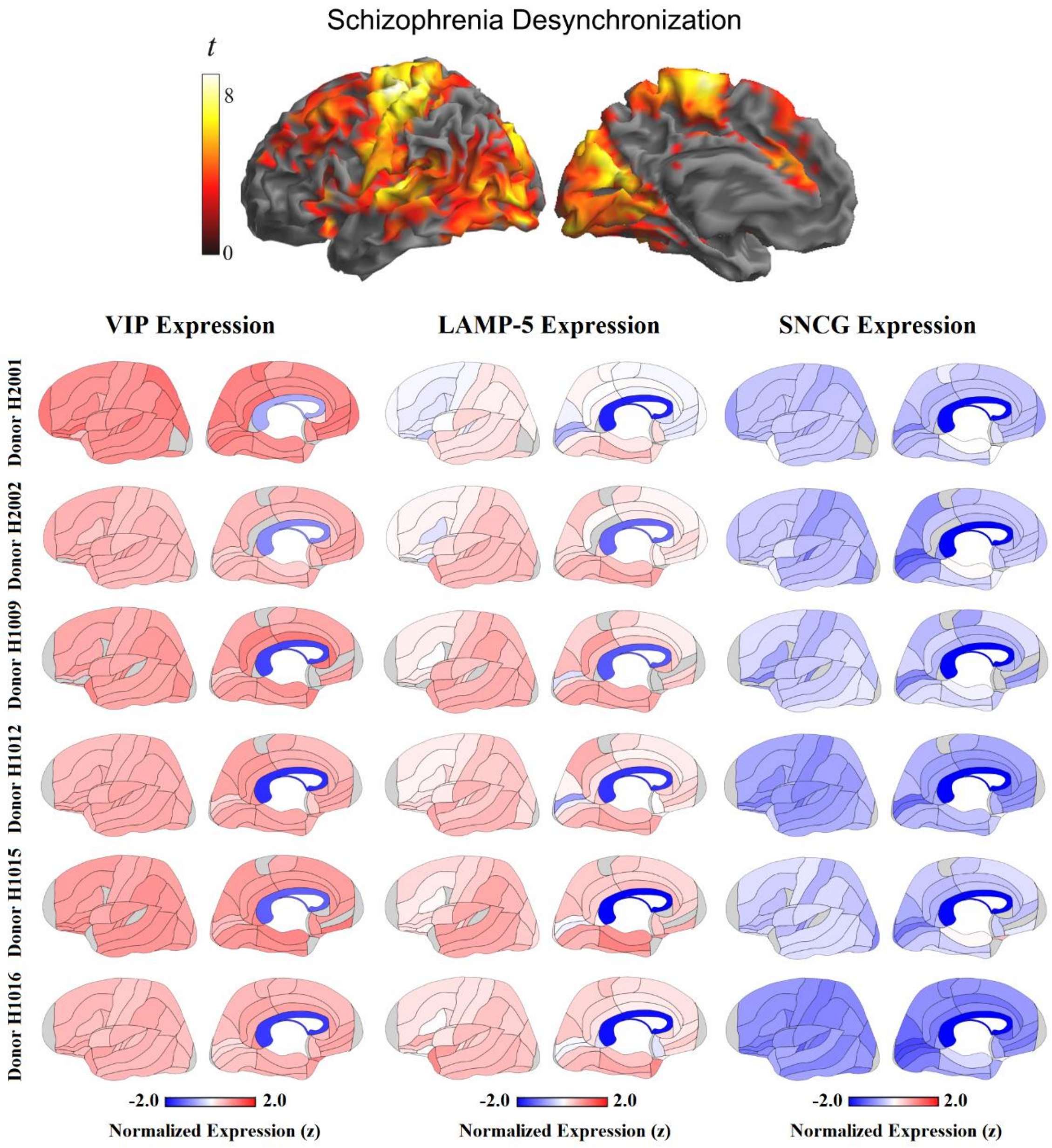

Supplementary Figure 3. Alterations in the local synchrony of the cerebral cortex identified in our study in patients with schizophrenia (top) and cortical expression of the three major GABA interneuron subclasses developmentally originated from the caudal ganglionic eminence of the subpallium. Note the poor resemblance between our findings and the distribution of these GABA interneurons indicating a level of specificity of the interneuron type putatively implicated. VIP, Vasoactive Intestinal Peptide. LAMP-5, Lysosomal-Associated Membrane Protein family (member 5). SNCG, Synuclein Gamma. Adapted with permission from the Allen Human Brain Atlas [https://human.brain-map.org/]).

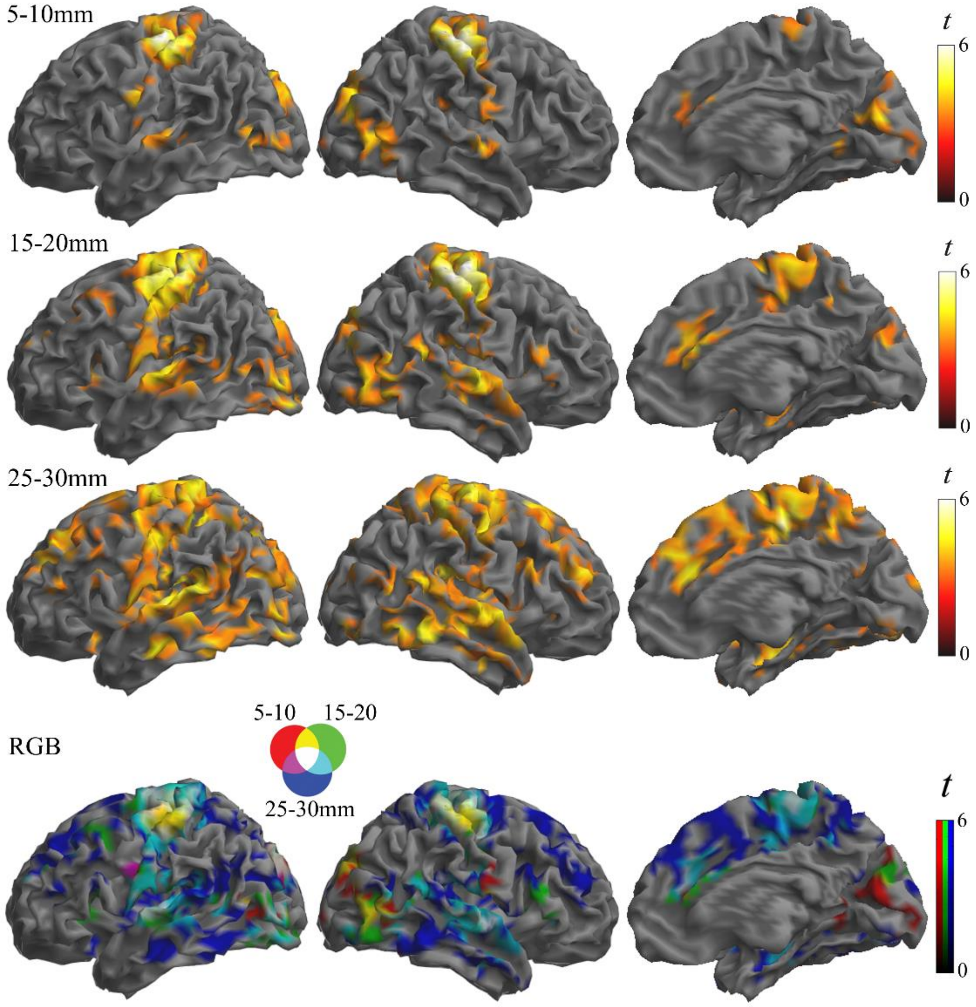

Supplementary Figure 4. Single effects illustrating differences between groups in IDAC measures at the three functional connectivity distances and the composite RGB (red, green and blue) display. All results were in the direction of patients showing weaker functional connectivity than control subjects.

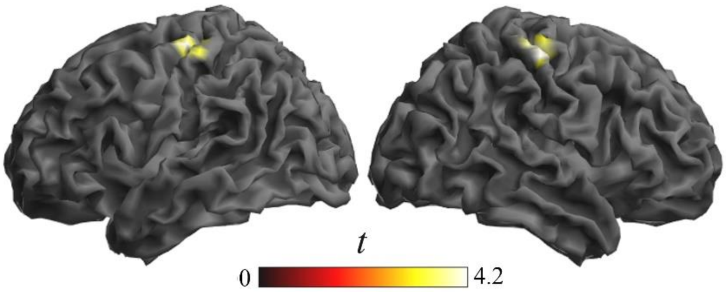

Supplementary Figure 5. Group-by-distance interaction. The images illustrate the regions showing weakening in local functional connectivity in patients significantly larger in the short-distance map (5-10 mm) than in the long-distance map (25-30 mm).

Supplementary Tables

Supplementary Table 1. Functional MRI statistical results. Group differences

	Cluster-level		*Peak-level*		
	Voxels	$P_{FWE\text{-}corr}$	***x y z***	***F***	***p***
**MAIN EFFECT OF GROUP**					
**Left Hemisphere**	13208	< 1e-16			
Somatosensory Cortex			-41 -30 60	71.5	<4e-16
Auditory Cortex			-47 -24 6	61.9	1e-14
Visual Cortex			-5 -69 18	54.6	4e-13
Motor Cortex			-44 -18 60	80.5	<4e-16
Anterior Insula			-35 18 -3	31.7	3e-8
Frontal Operculum			-44 15 3	28.4	1e-7
Orbitofrontal Cortex			-26 18 -15	31.9	2e-8
Anterior Cingulate Cortex			-2 18 24	37.9	1e-9
Dorsal Prefrontal Cortex			-35 18 48	29.9	6e-8
Hippocampus			-26 -21 -12	19.9	9e-6
**Right Hemisphere**	12772	< 1e-16			
Somatosensory Cortex			40 -33 60	98.5	<4e-16
Auditory Cortex			61 -9 -3	56.4	2e-13
Visual Cortex			40 -87 21	57.6	1e-13
Motor Cortex			34 -18 66	82.2	<4e-16
Anterior Insula			43 9 -12	34.1	8e-9
Frontal Operculum			55 33 15	28.4	1e-7
Orbitofrontal Cortex			40 24 -21	17.9	3e-5
Anterior Cingulate Cortex			7 30 24	42.5	1e-10
Dorsal Prefrontal Cortex			31 48 24	23.8	1e-6
Hippocampus			31 -21 -15	32.7	2e-8
**GROUP BY DISTANCE INTERACTION**					
Left Sensorimotor Cortex	77	0.003	-44 -21 60	9.4	9e-5
Right Sensorimotor Cortex	122	0.0001	46 -24 57	9.2	0.0001

$P_{FWE\text{-}corr}$, P (Family-Wise Error-corrected), whole brain. x y z, coordinates in Montreal Neurological Institute space.

Supplementary Table 2. Sensitivity analysis of group differences (covaried by sex, age and scanner)

	Non-covaried			*by Sex*		*by Age*		*by Scanner*	
**MAIN EFFECT OF GROUP**	***x y z***	***F***	***p***	***F***	***p***	***F***	***p***	***F***	***p***
**Left Hemisphere**									
Somatosensory Cortex	-41 -30 60	71.5	<4e-16	71.4	<4e-16	71.9	<4e-16	75.3	<4e-16
Auditory Cortex	-47 -24 6	61.9	1e-14	61.8	2e-14	65.1	4e-15	64.4	5e-15
Visual Cortex	-5 -69 18	54.6	4e-13	54.7	4e-13	58.9	5e-14	59.8	3e-14
Motor Cortex	-44 -18 60	80.5	<4e-16	80.6	<4e-16	82.3	<4e-16	84.5	<4e-16
Anterior Insula	-35 18 -3	31.7	3e-8	31.6	3e-8	34.9	6e-9	32.5	2e-8
Frontal Operculum	-44 15 3	28.4	1e-7	28.3	1e-7	33.4	1e-8	28.7	1e-7
Orbitofrontal Cortex	-26 18 -15	31.9	2e-8	31.9	2e-8	35.9	4e-9	33.2	1e-8
Anterior Cingulate C.	-2 18 24	37.9	1e-9	37.8	1e-9	40.8	3e-10	38.6	9e-10
Dorsal Prefrontal C.	-35 18 48	29.9	6e-8	30.2	6e-8	31.5	3e-8	31.2	3e-8
Hippocampus	-26 -21 -12	19.9	9e-6	19.8	9e-6	22.2	3e-6	20.4	8e-6
**Right Hemisphere**									
Somatosensory Cortex	40 -33 60	98.5	<4e-16	98.6	<4e-16	100	<4e-16	106	<4e-16
Auditory Cortex	61 -9 -3	56.4	2e-13	56.3	2e-13	59.4	5e-14	57.6	1e-13
Visual Cortex	40 -87 21	57.6	1e-13	57.4	1e-13	59.9	4e-14	63.5	7e-15
Motor Cortex	34 -18 66	82.2	<4e-16	82.1	<4e-16	85.1	<4e-16	87.4	<4e-16
Anterior Insula	43 9 -12	34.1	8e-9	34.2	8e-9	37.6	1e-9	34.7	8e-9
Frontal Operculum	55 33 15	28.4	1e-7	28.4	1e-7	29.2	9e-8	29.9	8e-8
Orbitofrontal Cortex	40 24 -21	17.9	3e-5	17.8	3e-5	20.4	7e-6	18.3	2e-5
Anterior Cingulate C.	7 30 24	42.5	1e-10	42.4	1e-10	47.9	1e-11	45.2	5e-11
Dorsal Prefrontal C.	31 48 24	23.8	1e-6	23.6	1e-6	25.1	7e-7	25.0	7e-7
Hippocampus	31 -21 -15	32.7	2e-8	32.7	2e-8	34.2	8e-9	33.3	1e-8
**GROUP BY DISTANCE INTERACTION**									
Left Sensorimotor C.	-44 -21 60	9.4	9e-5	9.5	8e-5	9.4	9e-5	10.0	5e-5
Right Sensorimotor C.	46 -24 57	9.2	0.0001	9.2	0.0001	9.2	0.0001	9.8	6e-5

x y z, coordinates in Montreal Neurological Institute space.

Supplementary Table 3. Functional MRI statistical results. Correlation analysis

	Cluster-level		*Peak-level*		
**NEGATIVE SYMPTOMS**	***Voxels***	***$P_{FWE\text{-}corr}$***	***x y z***	***F/t***	***p***
**Main Effect Across Distances**				***F***	
Left Visual Cortex	89	0.001	-53 -78 9	10.7	4e-5
Left Anterior Cingulate Cortex	50	0.033	-8 39 12	11.6	2e-5
Right Visual Cortex	67	0.010	61 -66 15	12.2	9e-6
Right Anterior Cingulate Cortex	71	0.007	7 45 15	12.3	8e-6
**Negative correlations**					
**Short Distance Map (5-10 mm)**				***t***	
Left Visual Cortex	168	0.0001	-47 -84 3	4.7	3e-6
Right Visual Cortex	87	0.016	55 -72 15	4.6	3e-6
Right Visual Cortex	73	0.036	10 -99 12	4.3	1e-5
Right Anterior Cingulate Cortex	95	0.010	7 45 12	3.2	0.0008
**Middle Distance Map (15-20 mm)**					
Left Visual Cortex	90	0.010	-47 -84 3	4.3	1e-5
Left Anterior Cingulate Cortex	114	0.002	-8 36 6	4.4	1e-5
Right Anterior Cingulate Cortex	94	0.011	13 36 3	3.7	0.0001
**POSITIVE SYMPTOMS**					
**Main Effect Across Distances**				***F***	
Left Motor Cortex (medial)	64	0.009	-5 -18 69	9.8	8e-5
Left Prefrontal Cortex	117	0.0001	-8 30 57	9.6	9e-5
**Correlation Interaction Short Distance < Long Distance**				***t***	
Left Motor Cortex (medial)	226	9e-6	-2 -15 66	4.4	1e-5
Left Prefrontal Cortex	203	2e-5	-8 30 57	4.1	2e-5
**Positive correlations**					
**Long Distance Map (25-30 mm)**					
Left Lateral Frontal Cortex	114	0.002	-41 39 30	3.8	0.0001
Left Anterior Insula	117	0.002	-26 27 3	4.4	7e-6
Right Homologue Broca's Area Region	542	2e-10	61 6 18	4.3	1e-5
Right Supramarginal Gyrus	99	0.008	55 -42 30	4.2	2e-5

$P_{FWE\text{-}corr}$, P (Family-Wise Error-corrected), whole brain. x y z, coordinates in Montreal Neurological Institute space.
